# Supplementary material for: Effects of 5-Year Nitrogen Addition on Species Composition and Diversity of an Alpine Steppe Plant Community on Qinghai-Tibetan Plateau
Source: Plants (Basel). 2022 Apr 1;11(7):966. doi: 10.3390/plants11070966 (PMC9002499; doi:10.3390/plants11070966)
Supplement: Supplementary file 1 [file plants-11-00966-s001.zip › plants-1632369-supplementary.pdf]

Table S1 The changes of total plant species number /m^2 under different N addition rates.

Treatment	total plant species number / m^2
CK1	5
CK2	10
CK3	6
N_I1	7
N_I2	9
N_I3	10
N_{II}1	4
N_{II}2	5
N_{II}3	9
N_{III}1	7
N_{III}2	5
N_{III}3	7
N_{IV}1	4
N_{IV}2	3
N_{IV}3	4
N_V1	5
N_V2	2
N_V3	4

Abbreviations: CK, N_I, N_{II}, N_{III}, N_{IV} and N_V are 0, 8, 24, 40, 56 and 72 kg N ha^{-1}·y^{-1}, respectively; similarly for the following tables and figures.

Table S2 The changes of total species cover under different N addition rates

Treatment	Total species cover / %
CK	83.00 ± 1.73 c
N_I	87.33 ± 1.20 b
N_{II}	90.67 ± 0.67 ab
N_{III}	92.00 ± 1.16 a
N_{IV}	93.67 ± 1.33 a
N_V	94.67 ± 0.88 a

Note: Data in the table are presented as mean ± standard error (SE). Different lowercase letters indicate significant differences between levels ($P < 0.05$).

Table S3 The importance values of all species of alpine steppe under different N addition rates

Specie name	CK	NI	N_{II}	N_{III}	N_{IV}	N_{V}
Leymus secalinus	0.50 ± 0.06 c	0.42 ± 0.05 c	0.56 ± 0.13 bc	0.73 ± 0.01 ab	0.78 ± 0.03 a	0.89 ± 0.04 a
Poa crymophila	0.33 ± 0.06 ab	0.39 ± 0.044 a	0.15 ± 0.09 bc	0.17 ± 0.01 bc	0.12 ± 0.07 c	0.09 ± 0.03 c
Agropyron cristatum	0.01 ± 0.01 b	0.03 ± 0.01 ab	0.07 ± 0.01 a	0.05 ± 0.01 ab	0.06 ± 0.03 ab	0.01 ± 0.01 b
Stipapurpurea	0.02 ± 0.02 a	0.02 ± 0.01 a	0.14 ± 0.14 a	0.01± 0.00 a	0.01 ± 0.01 a	—
Carex capillifolia	0.01 ± 0.01 a	0.01 ± 0.00 a	0.01 ± 0.01 a	0.01 ± 0.00 a	—	—
Carex melanantha	—	0.01 ± 0.00	—	—	—	—
Aster alpinus	0.08 ± 0.02 a	0.06 ± 0.02a	0.01 ± 0.01 b	0.01 ± 0.01 b	—	0.01 ± 0.00 b
Potentilla multifida	0.01 ± 0.00 a	0.01 ± 0.00 a	0.05 ± 0.04 a	0.01 ± 0.00 a	—	—
Thermopsis lanceolata	0.03 ± 0.030 a	—	—	0.01 ± 0.01 a	0.02 ± 0.02 a	—
Artemisia eriopoda	0.01 ± 0.00	0.01 ± 0.01	—	—	—	—
Dracocephalum heterophyllum	0.01 ± 0.00 a	—	0.01 ± 0.00 a	0.01 ± 0.01 a	0.01 ± 0.01 a	—
Aconitum gymnandrum	0.01 ± 0.01 a	0.01 ± 0.00 a	—	—	—	0.01 ± 0.00 a
Taraxacum mongolicum	0.01 ± 0.00 a	0.01 ± 0.01 a	0.01 ± 0.00 a	0.01± 0.00 a	—	—
Lancea tibetica	0.01 ± 0.00	—	—	—	—	—
Plantago asiatica	—	0.01 ± 0.00	—	—	—	—
Artemisia scoparia	—	0.04 ± 0.04	0.01 ± 0.01	—	—	—

Note: Data in the table are presented as mean ± standard error (SE) (n = 3). Different lowercase letters within the same row indicate significant differences among levels ($P < 0.05$). The "—" indicates that the species absented at this N addition rate level.

Table S4 The changes of soil physicochemical under different N addition rates

soil	CK	N_I	N_{II}	N_{III}	N_{IV}	N_V
TN g/kg	2.39±0.19 ab	2.31±0.12 b	2.26±0.22 b	2.88±0.15 a	2.23±0.16 b	2.62±0.07 ab
TC g/kg	31.6±0.31 b	32.52±0.64 b	32.92±0.42 b	32.50±1.08 b	33.56±0.61 b	37.37±1.06 a
TP g/kg	0.71±0.01 bc	0.73±0.00 ab	0.71±0.01 c	0.72±0.01 abc	0.73±0.00 ab	0.74±0.01 a
TK g/kg	20.74±0.30 a	20.80±0.30 a	20.44±0.14 a	20.38±0.16 a	20.76±0.06 a	20.73±0.15 a
NO_3-N g/kg	0.63±0.09 a	0.72±0.05 a	0.70±0.01 a	0.62±0.10 a	0.67±0.04 a	0.78±0.09 a
NH_4-N g/kg	0.90±0.13 c	0.91±0.04 c	1.11±0.06 bc	1.10±0.09 bc	1.30±0.07 ab	1.49±0.04 a
AP g/kg	0.06±0.01 a	0.02±0.00 b	0.02±0.00 b	0.03±0.00 ab	0.04±0.01 ab	0.03±0.01 b
AK g/kg	8.87±1.48 a	6.52±0.50 ab	5.01±0.36 b	5.65±0.70 b	5.91±0.33 b	5.93±0.65 b
Ca g/kg	37.85±0.35 b	40.99±0.87 ab	40.83±2.16 ab	42.06±1.54 ab	43.89±0.52 a	41.45±1.13 ab
Mg g/kg	12.51±0.02 a	12.65±0.12 a	12.48±0.06 a	12.58±0.07 a	12.45±0.05 a	12.71±0.11 a
S g/kg	0.46±0.00 a	0.46±0.00 a	0.43±0.00 b	0.45±0.00 a	0.45±0.00 ab	0.46±0.01 a
pH	7.83±0.08 a	7.98±0.05 a	7.92±0.07 a	7.85±0.05 a	7.83±0.07 a	7.94±0.14 a

Note: Data in the table are presented as mean ± standard error (SE). Different lowercase letters within the same row indicate significant differences among levels ($P < 0.05$)

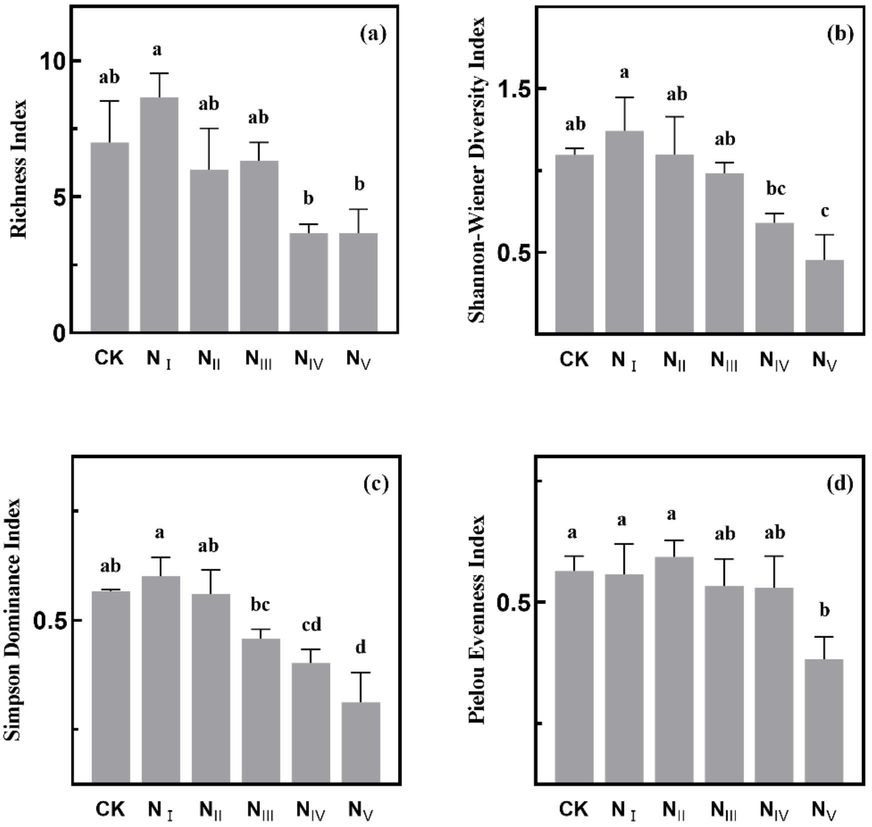

Figure S1. Richness index (a), Shannon-Weiner diversity index (b), Simpson dominance index (c), Pielou Evenness index (d) of alpine steppe under different N addition levels. Different lowercase letters indicate significant differences among levels ($P < 0.05$). Error bars represent standard error (SE) ($n = 3$).

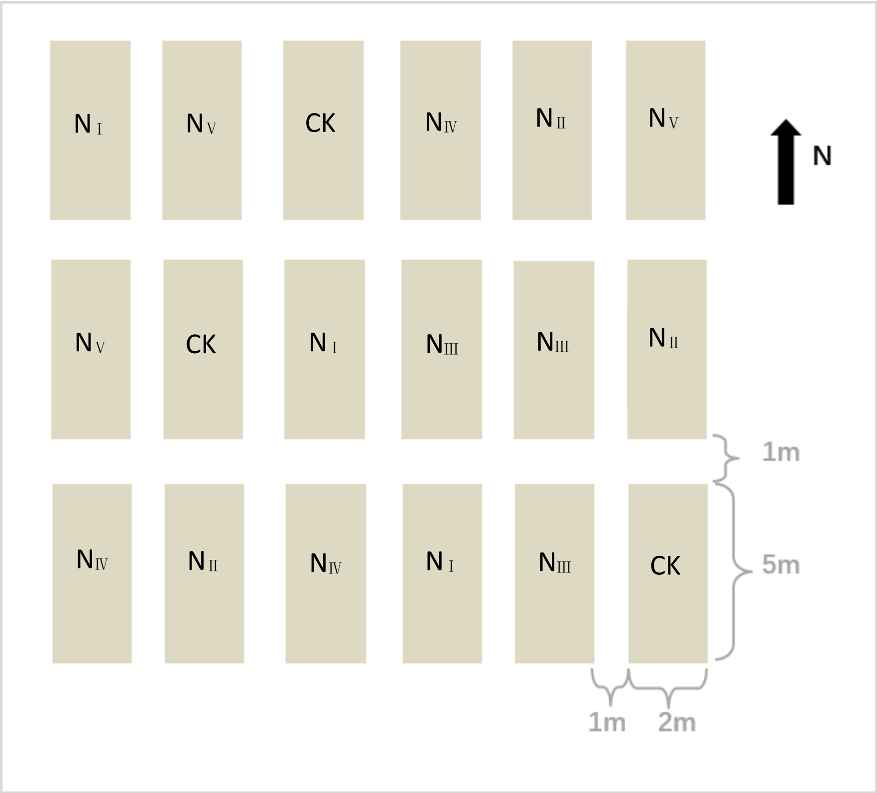

Figure S2 The arrangement of experimental plots of different N addition rates.
